# King Edward's Hospital Fund for London

**Published:** 1910-12-17

**Authors:** 


### KING EDWARD'S HOSPITAL FUND FOR LONDON.

### DISTRIBUTION OF GRANTS.

A meeting of the Governors and General Council of King Edward's Hospital Fund for London, for the purpose of awarding grants to the Hospitals, Convalescent Homes, and Consumption Sanatoria, for the present year, was held on Wednesday morning at St. James's Palace, His Serene Highness the Duke of Teck being in the Chair.

Those present were: Viscount Iveagh and The Speaker of the House of Commons (The Rt. Hon. James W. Lowther, M.P.); The Rev. T. Bowman Stephenson, D.D.; The Chief Rabbi (The Rev. Dr. Adler), Lord Sandhurst, Lord Revelstoke, The President of the Hospital Sunday and Hospital Saturday Funds (The Rt. Hon. The Lord Mayor), The Governor of the Bank of England (Mr. Reginald E. Johnston), The President of the Royal College of Physicians (Sir Thomas Barlow, Bart.), The President of the Royal College of Surgeons (Mr. Henry T. Butlin), the Rt. Hon. Sir Savile Crossley, Bart. (Hon. Secretary); The Rt. Hon. Sir Joseph Dimsdale, Bart.; The Rt. Hon. Sir Edgar Speyer, Bart.; Lt.-Col. The Rt. Hon. Sir Arthur Bigge; Sir Trevor Lawrence, Bart.; Sir W. S. Church, Bart.; Sir Julius Wernher, Bart.; Sir Henry Burdett, Sir Horace Marshall, Sir W. J. Collins, Sir Cooper Perry, Sir John Craggs, Mr. J. Danvers Power, Dr. Edwin Freshfield, Mr. Frederick M. Fry (Hon. Secretary), Mr. John G. Griffiths, Mr. Wiliam Latham, K.C., Professor W. Osler, and Mr. Albert G. Sandeman.

The minutes of the last meeting having been read and confirmed, His Serene Highness the Duke of Teck said: My Lords and Gentlemen,—This is the first distribution meeting of the Council since the sad event in May last, when the Fund suffered such an irreparable loss by the death of the Founder and Patron whose name it bears. The resolution, which was passed by the Executive Committee at the time, and which has been placed on record in the minutes which have just been read, expresses both the profound sorrow of the Members of this Council and the feelings of loyalty and gratitude with which they look back upon the work which his late Majesty did for the Fund and the Hospitals of London.

You have heard the gracious letter of acknowledgment, which is also on the minutes, from his present Majesty, who for many years, when Prince of Wales, presided not only over the meetings of this Council but over the work of the Fund in all its details, and who has since been graciously pleased to become its Patron.

I cannot proceed with the business of this meeting without adding a few words on behalf of myself and my colleagues, Lord Iveagh and the Speaker of the House of Commons. We have been appointed by his Majesty, on the recommendation of the Lord Chancellor, the Prime Minister, and the Governor of the Bank of England, in accordance with the Act of Parliament, to the responsible position of Governors of the Fund, and we would express our profound sense of the loss the Fund has sustained, and our intention, so far as in us lies, to endeavour humbly to follow the high tradition which has been established by his late and his present Majesty, until the time comes for the appointment of a new Royal President.

### The King's Interest.

I have now the honour to read to you, on behalf of the Governors, the following letter which has been addressed to us by Sir Arthur Bigge, by direction of his Majesty the King:—

"Buckingham Palace, 12th December, 1910.

"Dear Sirs,—I am commanded by the King to ask you to convey to the Council of King Edward's Hospital Fund for London, at the Distribution Meeting on Wednesday next, the expression of his Majesty's unabated interest in all that concerns the welfare of the Fund, over which he presided for nine years.

"His Majesty, recognising that the prosperity of the Fund is in no small measure due to the manner in which the Members of the Council and of the committees and the honorary officials have applied themselves to the administration of the money which the generosity of the public has provided, wishes to express his appreciation of the services which they have rendered to the sick poor, and his earnest hope for the continued success of their efforts.—Yours very truly, (Signed) "Arthur Bigge.

"To the Governors of King Edward's Hospital Fund for London."

His Serene Highness then said: My Lords and Gentlemen,—I propose that a vote of humble thanks be tendered to his Majesty for his gracious letter and for the continued interest which he has taken in the working of the Fund.

The Speaker of the House of Commons said: Your Serene Highness, my Lords and Gentlemen,—I rise to second the motion which you, sir, have just made, and I am sure that it needs no further words from me to add to those which you have just spoken. We are all so thoroughly acquainted with the deep and abiding interest which his Majesty has always taken in this Fund, that we are thankful to him for his gracious expressions, and we sincerely hope that he will continue, as we are assured he will, to feel that interest in the future which he has so unmistakably shown in the past in the proceedings of this Council and of the Fund. I have much pleasure in seconding the resolution.

The resolution was carried unanimously.

Sir Savile Crossley reported letters of regret from the following: The Lord-Lieutenant of the County of London (the Duke of Fife), the Bishop of London, the Bishop of Southwark, the Earl of Bessborough, Lord Rothschild, Lord Strathcona and Mount Royal, Lord Farquhar, the Master of Elibank, M.P., the Postmaster-General (the Right Hon. Herbert Samuel, M.P.), the Right Hon. Sydney Buxton, M.P., the President of the Local Government Board (the Right Hon. John Burns, M.P.), the Chairman of the County Council (Mr. W. Whitaker Thompson), Sir Everard Hambro, and Mr. J. Pierpont Morgan, jun.

### The Financial Statement.

In the absence of Lord Rothschild (Hon. Treasurer), Lord Revelstoke reported that the amount received by the Fund to December 10, after payment of expenses, was £146,584 10*s*.

The Fund had also received an interesting legacy during the year in the residue of the estate of Mr. Matyear, consisting of market garden property in Fulham, which was being put up for sale that very day.

Sir Henry Burdett stated that these were difficult times for an organisation like the League of Mercy—the members of which consisted of the most serious and thrifty members of the community. Further, they had had, in the golden periods of the year, a twofold interference with all the work of voluntary effort which was most unusual, almost unique, in the history of this country. He was therefore not in a position that day to state precisely the sum that the League would be able to give. But, having gone carefully into the figures, he had every confidence that, when the time arrived for drawing the cheque, the Governors and Council of this Fund would have good reason to be satisfied with the amount.

The Chairman of the Distribution Committee (Sir William Church) read the report of that committee as follows :—

Report of the Distribution Committee.

1. The Governors and General Council having this year placed the sum of £150,000 in the hands of the committee with a view to distribution amongst the London Hospitals, as against £147,000 in 1909, the committee beg to report as follows :—

2. The number of hospitals applying for grants is 106, as against 104 last year.

3. With regard to the Throat, Nose, and Ear Hospitals, the committee recommend that the policy of setting aside grants towards amalgamation in place of separate awards to any of the four institutions concerned should still be continued, and they have voted £2,000 this year to this purpose. No general scheme of amalgamation has been submitted by the hospitals during the year for the consideration of the Fund, and the committee would have recommended that the grant of £3,000 made in 1908 should lapse in accordance with the condition attaching to grants which remain for two years unapplied, had they not been informed that the possibility of a practicable arrangement between certain of the hospitals was under consideration. They accordingly recommend that the grant of £3,000 made in 1908 should be renewed for this year. The total sum held in reserve, in respect of annual grants, towards a general scheme of amalgamation now amounts to £7,500 in addition to the capital grant, amounting under certain conditions to at least £10,000, promised by the Fund.

4. The following grants made in 1908 for special purposes and retained in accordance with the practice of the Fund still remain unapplied : General Lying-in Hospital, £100 to surgical equipment; London Lock Hospital, £1,500, being the balance of a grant to the rebuilding of the Out-patient Department in Dean Stret; Miller General Hospital, £989, being the balance of a grant to extension; West End Hospital for Diseases of the Nervous System, £1,000, granted to rebuilding, subject to the scheme being submitted and approved; Wimbledon Cottage Hospital, £50 to improvements.

The committee recommend that the unpaid grants of the General Lying-in Hospital, the London Lock Hospital, the Miller General Hospital, and the Wimbledon Cottage Hospital should be renewed. In the case of the West End Hospital the committee are informed that the proposal before the Fund in 1908 for the rebuilding of the hospital on the site acquired for the purpose in Bulstrode Street has been abandoned, and that the site is for sale. No alternative scheme has yet been submitted, and the committee recommend that the grant should therefore lapse.

5. The committee have again had before them several schemes for improvements or extension submitted by hospitals in accordance with the resolution of the General Council to that effect. To some of these the committee have raised no objection, while in other cases they have been able to make suggestions which have been welcomed by the hospital authorities. It will be seen from the list of awards that in many cases grants in aid of schemes passed by the committee have been recommended.

6. The committee have as usual taken care that the needs of the poorer districts in the outlying parts of London should receive their due share of consideration, and that allowance should be made, where necessary, for the difficulty of maintaining adequate hospital accommodation in these parts. In addition to maintenance grants on the scale determined by the circumstances of the various hospitals, the committee have recommended capital grants in several cases where schemes of improvement have been sanctioned. Amongst these are final grants of £1,000 to the rebuilding of St. Mary's Hospital for Women and Children, Plaistow, and £5,000 to the extension of the West Ham Hospital, which will enable both these schemes to be completed, and the much-needed accommodation rendered available at the earliest possible moment, free of debt.

7. While regretting the failure of the negotiations for the substitution of a single scheme for the rebuilding proposals of the hospitals at North Wimbledon and South Wimbledon, the committee recommend that a building grant should be made to each institution of the amount indicated in the list of awards.

8. The sum of £5,000 voted towards the removal of King's College Hospital to South London increases the total amount contributed by the Fund to this object to £37,000.

Convalescent Consumption Sanatoriums.

9. The committee are glad to learn that a scheme has been prepared by the Convalescent Homes Committee for rendering a certain number of beds in consumption sanatoria in the country available for patients direct from London hospitals, more especially from consumption hospitals which have no country branches of their own. The Distribution Committee have always viewed with special satisfaction the facilities possessed by consumption hospitals which have country branches for the transfer of suitable cases to surroundings more favourable to recovery; and they welcome any practicable method by which more of such accommodation can be rendered available without charging the funds of a hospital with the necessity of providing for a large capital expenditure.

Hospitals and Medical Schools.—The Case of St. George's.

10. In the case of St. George's Hospital, the committee have had under their special consideration the appearance, in the general accounts of the hospital for 1909, of certain payments to the school which were stated to be for work done for the hospital, and which were formerly paid out of a discretionary fund. The inquiries made by the committee as to the legitimacy of this change confirmed the statement in the annual report of the hospital that the payments in question were made on account of definite work done, chiefly pathological and bacteriological examinations, in connection with the actual treatment of patients. That is, the payments were for the kind of work which is described in Appendix III. of the report of Sir Edward Fry's committee as work which would have to be paid for in any case, and which would have to be taken into account in assessing the payments and receipts on either side.* Such work, moreover, differs entirely from the general benefits, described in paragraphs 13 to 22 of the report as being conferred by medical schools on hospitals, and as being fairly set off against the benefits conferred by the hospitals on the schools. The opinion of the Fund, that, on the principles laid down by Sir Edward Fry's com-

* See note cn page 363.

mittee, specific payments for such definite work done in respect of the treatment of patients, whether made to the school or to any other agency, could be charged to the general funds of the hospital, while no other payments to the school could be so charged, was notified to St. George's, in reply to a question from the hospital, by the executive committee in March 1909. But on looking into the method by which the amount fairly chargeable to the hospital on account of this work had been arrived at, the Distribution Committee came to the conclusion that the apportionment of the expenses of the laboratories as between the treatment of patients and medical education required investigation, and they have invited the hospital to submit further details in order that the committee may satisfy themselves that the payment made to the school bears a proper proportion to such definite work done for the hospital.

In the meantime they recommend that the grant to the hospital this year should be retained until the Fund is satisfied that the financial relations between hospital and school are in accordance with the principles intimated by the Fund in March 1909, and that the general account of the hospital in respect of 1909, as well as 1910, is clear from any payment on account of medical education.

11. The reports of the visitors have again proved of the greatest value to the committee, and many of their observations have been brought to the notice of the hospital authorities. The committee have also continued the practice of drawing the attention of the hospitals concerned to cases where the excess of cost above the average, as disclosed by the Fund's statistical report, seems to call either for specific justification or for greater attention to economy.

The details of the awards to the various hospitals are appended.

For the Committee,
W. S. Church, *Chairman.*

Sir Savile Crossley (Hon. Secretary) presented the list of awards as follows :—

Grants Recommended by the Distribution Committee.

The Distribution Committee desire to draw attention to the fact that the absence of a grant does not necessarily imply dissatisfaction.

Alexandra Hospital for Children.—£400.
Beckenham Cottage Hospital.—£50; of which £25 to renewal of floors.
Belgrave Hospital.—£1,000; to reduce building debt.
Blackheath and Charlton Cottage Hospital.—£100.
Bolingbroke Hospital.—£1,500.
British Lying-in Hospital.—£400.
Catholic Nursing Institute.
Central London Ophthalmic Hospital.—£1,000; to rebuilding in accordance with scheme submitted to the Fund.
Central London Throat and Ear Hospital.—See paragraph 3 of Report.
Charing Cross Hospital.—£3,000.
Chelsea Hospital for Women.—£750.
Cheyne Hospital.
City of London Hospital for Diseases of the Chest, Victoria Park.—£4,000.
City of London Lying-in Hospital.—£500.
Clapham Maternity Hospital.—£200.
Dreadnought Hospital.—£4,000; of which £1,000 to reduce debt.
Ealing Cottage Hospital.—£500; to rebuilding in accordance with scheme submitted to the Fund.
East End Mothers' Lying-in Home.—£500; of which £250 to reduce debt.
East London Hospital for Children.—£2,000; of which £250 to improvements.
Eltham and Mottingham Cottage Hospital.—£100.
Evelina Hospital.—£150.
French Hospital.—£200.
Friedenheim Hospital.—£50.
General Lying-in Hospital.—£250.
German Hospital.—£200.
Gordon Hospital for Fistula, etc.—£25.
Great Northern Central Hospital.—£4,000; of which £1,000 to reduce debt.
Grosvenor Hospital for Women.—£350.
Guy's Hospital.—£7,500; of which £1,500 to building improvements in accordance with scheme submitted to the Fund.
Hampstead General Hospital.—£3,150; of which £1,500 to reduce debt and £1,650 on account of maintenance and rent, as agreed upon amalgamation with North-West London Hospital.
Home and Infirmary for Sick Children.—£250; of which £50 to outside staircase.
Home for Mothers and Babies, Woolwich.—£100.
Hospital and Home for Incurable Children, Hampstead.—£100; of which £75 to improvements.
Hospital for Consumption, Brompton (including Sanatorium at Frimley).—£3,000.
Hospital for Diseases of the Throat.—See paragraph 3 of Report.
Hospital for Epilepsy and Paralysis.—£750.
Hospital for Invalid Gentlewomen.—£200.
Hospital for Sick Children.—£4,000; of which £1,500 to recent improvements in accordance with scheme submitted to the Fund.
Hospital for Women, Soho Square.—£1,000.
Hospital of St. John and St. Elizabeth.—£350.
Infants Hospital.—£25.
Italian Hospital.—£500.
Kensington and Fulham General Hospital (late Queen's Jubilee Hospital).
Kensington Dispensary and Children's Hospital.—£25.
King's College Hospital.—£7,000; of which £5,000 to removal fund.
Leyton, Walthamstow, and Wanstead Children's and General Hospital.—£250.
London Fever Hospital.—£250.
London Homœopathic Hospital.—£500; to rebuilding of Nursing Home in accordance with scheme submitted to the Fund.
London Hospital.—£12,000.
London Lock Hospital.—£1,500; to the Harrow Road Hospital, of which £1,000 to reduce debt.
London Temperance Hospital.—£1,500.
London Throat Hospital.—See paragraph 3 of report.
Maternity Charity and District Nurses' Home, Plaistow.—£100.
Medical Mission Hospital, Balaam Street, Plaistow.—£35.
Memorial Hospital, Mildmay Park.—£50.
Metropolitan Hospital.—£3,500; of which £1,000 to reduce debt on structural improvements carried out in accordance with scheme submitted to the Fund.
Middlesex Hospital.—£4,000.
Middlesex Hospital Cancer Charity.—The Committee are glad to see that no assistance from the Fund is required this year.
Mildmay Mission Hospital.—£400.
Miller General Hospital for South-East London.—£1,500; of which £750 to extension, subject to scheme being approved.
Mount Vernon Hospital for Consumption (including Sanatorium at Northwood).—£1,500.
National Anti-Vivisection Hospital.
National Dental Hospital.—£400; of which £150 to improvements in accordance with scheme submitted to the Fund.
National Hospital for Diseases of the Heart.
National Hospital for the Paralysed and Epileptic.—£1,000.
New Hospital for Women.—£1,500; of which £750 to building improvements in accordance with scheme submitted to the Fund.
Norwood Cottage Hospital.—£100.
Paddington Green Children's Hospital.—£1,750; of which £1,000 to reconstruction of out-patient department, subject to approval of scheme.
Passmore Edwards Acton Cottage Hospital.—£75.
Passmore Edwards East Ham Hospital.—£50.
Passmore Edwards Hospital for Willesden.—£25.
Passmore Edwards Cottage Hospital, Wood Green.—£25.
Poplar Hospital.—£400.
Prince of Wales's General Hospital.—£4,000; of which £2,000 to reduce debt.
Queen Charlotte's Lying-in Hospital.—£1,500; of which £500 to improvements.
Queen's Hospital for Children.—£3,000; of which £1,250 to reduce building debt.
Royal Dental Hospital of London.—£1,250; of which £250 to recent improvements.
Royal Ear Hospital.—See paragraph 3 of report.
Royal Eye Hospital.—£1,000; of which £250 to reduce debt.
Royal Free Hospital.—£4,000.

Royal Hospital for Diseases of the Chest, City Road.—£1,250; of which £250 to reduce debt.
Royal Hospital, Richmond.—£200; of which £50 to fire-escape balconies.
Royal London Ophthalmic Hospital.—£2,500.
Royal National Orthopædic Hospital.—£7,000; of which £250 as agreed upon amalgamation.
Royal Waterloo Hospital for Children and Women.—£1,500.
St. George's Hospital.—£2,000; to be retained until the Fund is satisfied that the relations between hospital and medical school are in accordance with the principles intimated by the Fund in March 1909. See paragraph 10 of report.
St. John's Hospital for Diseases of the Skin.—£600; of which £500 to reduce building debt.
St. John's Hospital, Lewisham.—£300.
St. Luke's House.—£50.
St. Mark's Hospital.—£400; of which £300 to improvements in nursing accommodation in accordance with scheme submitted to the Fund.
St. Mary's Hospital.—£6,500; of which £2,500 to reduce debt.
St. Mary's Hospital for Women and Children, Plaistow.—£1,500; of which £1,000 to enable the new building to be furnished and opened free of debt.
St. Monica's Hospital.—£100.
St. Peter's Hospital for Stone.—£150.
St. Saviour's Hospital, Osnaburgh Street.—£200.
St. Vincent's Home for Crippled Boys.
Samaritan Free Hospital.—£1,000.
Santa Claus Home.—£25.
South Wimbledon, Merton and District Cottage Hospital.—£1,000; to the rebuilding of the hospital under the name of the Wimbledon and Merton Hospital, in accordance with scheme submitted to the Fund.
University College Hospital.—£5,500; of which £1,500 to reduce debt.
Victoria Hospital for Children.—£1,000.
West End Hospital for Diseases of the Nervous System.—£250.
Western Ophthalmic Hospital.—£275; to reduction of debt on rebuilding of out-patient department.
West Ham and East London Hospital.—£5,500; of which £5,000 to building fund, to enable th new building to be opened free of debt.
West London Hospital.—£5,000; of whic £2,000 to reduce debt, on the understanding that special efforts are made to obtain increased support from the public.
Westminster Hospital.—£3,000.
Wimbledon Cottage Hospital.—£550; of which £500 to rebuilding in accordance with scheme submitted to the Fund.

Special Grant.

| | |
|---|---|
| Set aside for amalgamation of Throat and Ear Hospitals ... ... ... ... ... ... | £2,000 |

SUMMARY.

| | |
|---|---|
| Grants ... ... ... ... ... ... ... | £148,000 |
| Special grant set aside for amalgamation ... ... | 2,000 |
| Total grants to hospitals for the year 1910... | £150,000 |

The Chairman of the Convalescent Homes Committee (Mr. William Latham, K.C.) read the report of that committee as follows :—

Report of the Convalescent Homes Committee.

1. The Governors and General Council have this year placed the sum of £5,000 in the hands of the committee with a view to distribution amongst convalescent homes and consumption sanatoria. The amount distributed in 1909 was £4,000, which included £1,000 brought forward from 1908.

2. The number of applications eligible for consideration amounted to 45 from convalescent homes and 8 from consumption sanatoria, as against 37 and 4 respectively last year.

3. The committee have again received new applications from several convalescent homes, to most of which grants are recommended in consideration of the number of London patients admitted during the past year. At the same time the committee have adhered to their original policy of giving special consideration to the convalescent homes closely connected with London hospitals.

4. The increase in the number of consumption sanatoria applying for grants is very gratifying. An examination of the applications, however, revealed the fact that, as in previous years, the most urgent need, so far as London patients were concerned, was not for maintenance grants to supplement the income of existing institutions which have secured public confidence, but rather for an increase in the accommodation available without delay for hospital cases requiring immediate sanatorium treatment in the country. The committee are glad, therefore, to report that they have been able to make arrangements in the case of three sanatoria for beds to be placed at the disposal of London hospitals during the year 1911 in return for grants made by the Fund. The total number of beds thus secured amounts to eighteen, situated as follows: Two beds at the Kelling Sanatorium, Norfolk; six beds at the Northampton Sanatorium, Creaton, Northants; ten beds at the Devon and Cornwall Sanatorium, South Brent, Devon.

5. In recommending the allocation of these country beds to hospitals in London the committee have taken the view that preference should be given (in order of relative size) to the two Consumption Hospitals which have no country branches, and that the rest of the beds should be divided between the two largest of the general hospitals which apply to the Fund.

They accordingly recommend the following allocation :—Eight beds to the City of London Hospital for Diseases of the Chest, four beds to the Royal Hospital for Diseases of the Chest, three beds to the London Hospital, and three beds to Guy's Hospital.

The committee are of opinion that this allocation will afford at the same time the greatest benefit to the consumptive poor and the best method of testing the working of the scheme under varied conditions.

6. Should the scheme in its practical working be as successful as there is every reason to expect, the committee trust not only that further offers of beds on the same terms will be forthcoming, but that the Council will authorise them to consider, subject to proper safeguards, applications for capital grants to provide additional accommodation at existing sanatoria, to be placed at the disposal of London hospitals. This might well prove to be an economical method of supplementing the very valuable, but necessarily limited, accommodation which can be provided by means of country branches attached to London hospitals.

7. The committee are greatly indebted to the visitors who undertook the task of inspecting the sanatoria applying for grants, and who thus furnished the committee with invaluable evidence as to the suitability of the institutions in which beds have been secured.

The grants recommended to the various institutions are appended.

For the Committee,
William Latham, *Chairman.*

Mr. Frederick M. Fry (Hon. Secretary) presented the list of awards as follows :—

Grants Recommended by the Convalescent Homes Committee.

A.—CONSUMPTION SANATORIA.

The Convalescent Homes Committee desire to draw attention to the fact that the absence of a grant does not necessarily imply dissatisfaction.

Children's Sanatorium, Holt, Norfolk.—The Committee are glad to see that the balance of the building fund for a permanent Sanatorium has been provided by a private donor.
Devon and Cornwall Sanatorium, Didworthy, South Brent.—£875; in consideration of the reservation of ten beds during 1911 for the use of certain London hospitals. (See paragraphs 4 and 5 of report.)
Fairlight Sanatorium, Hastings.
Kelling Sanatorium, Holt, Norfolk.—£175; in consideration of the reservation of two beds during 1911 for the use of certain London hospitals. (See paragraphs 4 and 5 of report.)
Maitland Sanatorium, Peppard, Oxon.
National Association for the Establishment and Maintenance of Sanatoria for Workers, Benenden, Kent.—£250; to reduce building debt.
Northampton Sanatorium, Creaton, Northants.—£525; in consideration of the reservation of six beds during 1911 for the use of certain London hospitals. (See paragraphs 4 and 5 of report.)
Royal National Hospital for Consumption, Ventnor.

### B.—CONVALESCENT HOMES.

The names of convalescent homes receiving no grant are not published.

Alexandra Hospital for Children, Painswick.—£25.
All Saints' Convalescent Home for Children, Highgate.—£25.
Baldwin Brown Home for Convalescent Poor, Herne Bay.—£25.
Brentwood Convalescent Home for London Children, Brentwood.—£25.
Charing Cross Hospital, Limpsfield.—£200; of which £100 to reduce debt and £100 maintenance grant with a view to encouraging the admission without payment of patients direct from the hospital.
Chelsea Hospital for Women, St. Leonards.—£50; with a view to encouraging the admission without payment of patients direct from the hospital.
Children's Cottage Hospital, Cold Ash, Newbury.—£25; in consideration of convalescent cases.
Children's Home Hospital, Barnet.—£25; in consideration of convalescent cases.
Convalescent Police Seaside Home, Hove.—£50.
Deptford Medical Mission, Bexhill.—£25.
East London Hospital for Children, Bognor.—£250.
Erskine House, Hampstead Heath.—£25.
Great Northern Central Hospital, Clacton-on-Sea.—£100.
Home and Infirmary for Sick Children (Sydenham), Broadstairs.—£50.
Hospital for Sick Children (Great Ormond Street), Highgate.—£225.
King's College Hospital, Hemel Hempstead.—£100.
London Hospital, Tankerton.—£50.
Mary Wardell Convalescent Home for Scarlet Fever, Stanmore.—£25.
Metropolitan Convalescent Institution (Children), Broadstairs—£150.
Metropolitan Convalescent Institution, Walton.—£200.
Metropolitan Hospital, Cranbrook.—£50.
Middlesex Hospital, Clacton.—£400.
Mildmay Mission Hospital, Hendon.—£100.
Mrs. Kitto's Free Convalescent Home, Reigate.—£50.
National Hospital for the Paralysed, Finchley.—£100; with a view to facilitating the admission of patients without payment.
Paddington Green Children's Hospital, Slough.—£100.
Poplar Hospital, Walton-on-Naze.—£50.
Queen Charlotte's Lying-in Hospital, Kilburn.—£50.
St. Andrew's Convalescent Home, Folkestone.—£100.
St. Andrew's Convalescent Hospital, Clewer.—£50.
St. Mary's Home for Children, Broadstairs.—£50; in consideration of convalescent cases.
St. Michael's Home for Men and Women, Westgate-on-Sea.—£25.
Sunshine Home of Recovery, Hurstpierpoint.—£25.
Surgical Home for Boys, Banstead.—£25.
Victoria Home for Invalid Children, Margate.—£100; to building, in consideration of convalescent cases.
Victoria Hospital for Children (Chelsea), Broadstairs.—£225.
Winifred House Invalid Children's Convalescent Hospital Home, Tollington Park.—£25.

### SUMMARY.

| | |
|---|---|
| Grants to Consumption Sanatoria ... ... ... | £1,825 |
| Grants to Convalescent Homes ... ... ... ... | £3,175 |
| Total Grants recommended ... ... ... | £5,000 |

### The Duke's Speech.

His Serene Highness the Duke of Teck, in moving the adoption of the Reports and Awards, said:—

My Lords and Gentlemen,—I think that the Reports which have just been read, and the statement which has been made by Lord Revelstoke, afford gratifying evidence of the continued progress of the Fund.

The invested capital is now very considerable, and we are much indebted to the eminent men of business on the Finance Committee, who look after these investments for us. But the income from investments provides less than half the amount distributed, and for the balance we rely upon the support of the charitable public, including the contributions collected by the League of Mercy.

Among the amounts received during the year, I would mention, with special gratitude, the sum of £500 received from his Majesty, the Patron of the Fund, in augmentation, from £500 to £1,000, of the annual subscription he gave when President; and also the annual subscription of £100, with which his Royal Highness the Prince of Wales has signified his interest in the Fund. The receipts also include an annual subscription of £5,000 from Mr. Walter Morrison, and an anonymous donation of £10,000.

The amount distributed has again been increased. Last year we reached, for the first time, a total sum of £150,000. This year we give £150,000 to the Hospitals alone, making, with £5,000 to Consumption Sanatoria and Convalescent Homes, a total of £155,000. We have thus left behind our original goal of £150,000, and, in response to the necessarily increasing demands upon us, we have begun a fresh advance.

The Report of the Distribution Committee shows the care taken by the committee to allot the grants as far as possible in due relation to the circumstances and claims of the various hospitals, as revealed by the accounts, the visitors' reports, and the other evidence before the committee.

I would particularly draw attention to the grants for special purposes. In practically every case where such a grant is at all large, you will see that a scheme has been submitted by the hospital and carefully examined by the committee. This practice ensures that the Fund knows, and approves of, the manner in which the grant is going to be spent. The fact that so many special grants are recommended shows that the hospitals generally are not in the habit of proposing unreasonable schemes for locking up money in bricks and mortar, and that the Fund does not discourage reasonable schemes of extension or improvement.

You will have noticed that the Report calls special attention to the grants by which two hospitals, in the very poor districts in the far East of London, are enabled to open their new buildings free of debt.

You will also notice that questions have arisen during the past two years as to the exact method of applying the recommendations of Sir Edward Fry's Committee on the subject of Hospitals and Medical Schools. It is, and has always been, the intention of the Fund to carry out the recommendations of that committee in the letter and in the spirit. And you will, I am sure, be glad to see that the Distribution Committee is giving such careful attention to this matter, and that their attitude is based upon the exact terms of the Report of Sir Edward Fry's Committee.

The Report of the Convalescent Homes Committee shows that the scheme, which they originated, for placing beds at Consumption Sanatoria, by means of grants from the Fund, at the disposal of patients in London hospitals who urgently need country treatment, has now taken practical shape. A beginning can, if the Council approves, be made on January 1 next, with eighteen beds in three different sanatoria. It is proposed that most of these beds should be allotted to the two Consumption Hospitals which have no country branches of their own, and the remainder to two of the largest general hospitals. It was not possible to secure any more beds without making capital grants.

But the committee propose, if you adopt their Report, to consider applications for capital grants another year. If the scheme, on its present small scale, is successful, it might be thus enlarged. The committee would, of course, take due precautions to ensure that any arrangement made should be fair to London, while the sanatoria would doubtless see that it was fair to the localities for which they were originally founded.

Hospital Contracts.

The work of the Executive Committee is not the less important and responsible because it does not form the subject of a detailed report at this meeting. It has included this year a consideration of the question of hospital contracts. It has often been suggested that it would be an advantage for the hospitals of London to enter into joint contracts, and thus secure the advantages of purchasing on a larger scale than they now do as separate buyers. Some correspondents have considered the advantages so obvious, and the difficulties so insignificant, that they have severely criticised the hospitals for not having adopted the principle long ago. It so happens that last year a scheme was actually started in New York, and the Executive Committee thought that this would be a good opportunity to appoint a sub-committee to inquire into the subject, and ascertain whether the Fund could usefully take any action in the matter. This was done, with the approval of H.R.H. the President, and the report of the sub-committee, of which Mr. John G. Griffiths acted as Chairman, has recently been issued. I hope that the careful statement which it contains, of the possible advantages and difficulties of co-operative purchase by groups of independent voluntary hospitals, may be of assistance to the hospitals which are now trying this experiment, or which may be thinking of doing so; and that it may satisfy the public that the possibilities of economy in this direction have not been overlooked. The sub-committee has also prepared a schedule of specimen forms of tender for hospital contracts, and it is hoped that this also will prove to be of assistance to many of the hospitals in securing economical buying.

The particulars as to prices and cost of working, collected and circulated by means of the Statistical Report published annually by the Fund, have been of great use in connection with this inquiry. I am glad to see that these Statistical Reports, which have in the past produced such excellent results in the direction of economy, have again been enlarged, and now deal with the accounts of nearly one hundred hospitals.

Before moving the adoption of the Reports, I would express, on behalf of my fellow Governors and myself, our thanks to the members of the various Committees, and to the Honorary Officers, including the Visitors who have inspected the Hospitals and Consumption Sanatoria on behalf of the Fund. I may say that during the short time that we have occupied our present position, we have been impressed by the number of committee meetings whose minutes come before us, and by the variety of the business dealt with. They form a striking illustration of the way in which, under the supervision and encouragement of its two illustrious Presidents, the Fund has grown not only in financial magnitude, but also, still more remarkably, in the number and importance of its administrative functions. I feel sure that the Council and Committees will assist the Governors in the common effort to ensure that the Fund shall continue to receive, and to merit, the confidence of the King and of the public.

I now beg to move the adoption of the Reports.

The Chief Rabbi (the Rev. Dr. Adler), in seconding the adoption of the reports, said that he was sure that all members of the Council were impressed by the very valuable analysis with which his Serene Highness had favoured them. The value of this Fund consisted not merely in the financial help which it gave to the various struggling hospitals, but also in the most excellent advice which the Fund gave as to the way in which the hospitals might be enabled to combine economy with efficiency, and the valuable hints in regard to remedying evils and introducing still further improvement. It was a source of gratification to him to know that the efficiency of the various consumption sanatoria would be increased, and that next year it was intended to entertain applications for capital grants to these indispensably necessary institutions.

After discussion, the adoption of the reports was put and carried.

On the motion of Sir Trevor Lawrence, seconded by Sir John Craggs, it was resolved that the cheques for the grants should be posted on Tuesday, December 20, except in the case of grants for the reservation of beds in consumption sanatoria during 1911, which should be posted on January 3.

The Executive Committee having reported that it had been the custom at the Distributing Committees that a member should absent himself from the committee during any discussion of the affairs of an institution with which he was in any way identified, the Council, on the motion of Mr. John G. Griffiths, seconded by Lord Sandhurst, passed a resolution embodying this custom in a rule.

Arrangements for 1911.

His Serene Highness the Duke of Teck then said: My Lords and Gentlemen,—It was the practice of the President to announce at this meeting his intentions with regard to the appointment of the General Council and committees for the coming year. I will, therefore, state briefly the proposals of the Governors.

Before doing so I must allude to the great loss the Fund has sustained during the year in the death of Mr. Hugh C. Smith, who had served for so many years as a trustee, member of the Council, chairman of the Executive Committee, and latterly member of Finance Committee. We have also lost by death the very valuable services of Mr. John Langton as one of the visitors of the Fund.

With regard to the General Council, we propose to reappoint the whole of the members as at present, and the same with the Executive Committee and the Finance Committee. On the Distribution and Convalescent Homes Committees, we propose to reappoint the present members, except that Mr. Partington, who to his great regret has been prevented by ill-health from taking a full share in the very arduous work of the Distribution Committee, will join the Convalescent Homes Committee, as also will Sir William Bennett, and that Mr. Leonard Cohen will be appointed to the Distribution Committee. The visitors are appointed later on in the year.

The Out-Patients Committee.

Last March his Majesty the King, then President of the Fund, expressed the hope that the proposed Committee of Inquiry into the Out-patient question would be appointed before the close of this year, and would begin its work early in 1911. I have great pleasure in stating that we have been fortunate enough to secure the services of three eminent commissioners—Lord Mersey, who will be chairman of the committee, Lord Northcote, and the Bishop of Stepney.

You will remember that the President also indicated that the function of the proposed committee would be to inquire into the method prevailing in the London voluntary hospitals with regard to the admission of out-patients. The Executive Committee have recommended that this function should be expressed in the following terms of reference, which only await your confirmation :—

"To consider and report generally as to the circumstances and conditions under which patients are admitted to the casualty and out-patient departments of the London voluntary hospitals, and especially as to what precautions are taken to prevent the admission of persons who are unsuitable, and as to whether adequate provision is made for the admission of such persons as are suitable; and to make such recommendations as may seem to them desirable."

I trust that this inquiry will do much to settle this very important and much-discussed question.

I have now to ask you to consider the delegation resolutions which have been circulated, and which lie on the table. If they are approved, they will only need to be confirmed at the formal meeting which must be held for the purpose in the New Year.

On the motion of Sir Thomas Barlow, seconded by Sir John Craggs, a resolution was passed, delegating powers to the Finance, Distribution, and Convalescent Homes Committees.

On the motion of Lord Iveagh, seconded by Sir Joseph Dimsdale, a resolution was passed, delegating powers to the Out-patient Inquiry Committee.

On the motion of Mr. J. Danvers Power, seconded by Mr. Albert G. Sandeman, a resolution was passed, delegating powers to the Executive Committee.

His Serene Highness then moved a resolution as to the custody of the keys of the seal, which was seconded by the Governor of the Bank of England (Mr. Reginald E. Johnston), and carried.

The Lord Mayor (Sir Vezey Strong) proposed a vote of thanks to his Serene Highness for presiding. The resolution was seconded by Professor Osler, and carried unanimously.

The Duke of Teck, in acknowledging the resolution, said —I feel it a great honour to have been appointed one of the Governors of the Fund, and I trust that I shall be able worthily to fulfil the duties of that office.

The proceedings then terminated.

NOTE.—The following is an extract from this appendix, which is referred to by Sir Edward Fry's Committee (Report, par. 9), as "the third appendix, which we (the Distribution Committee, see p. 358, second column) adopt as part of our report":—"It must be remembered that, apart from these (*i.e.* the general services rendered by the schools to the hospitals), which are separately dealt with in the Report, par. 21, the schools, in most cases, undertake many specific laboratory examinations solely for the purpose of helping the physicians and surgeons to form correct diagnoses in the exclusive interests of the patients, and that these would have to be paid for by the hospitals in any case. Where such examinations are made in a laboratory entirely maintained by a school, it is obvious that, in any exact assessment of the payments and receipts, on either side, they would have to be taken into account."

